# Role of thrombopoietin receptor agonists in chemotherapy-induced thrombocytopenia: A meta-analysis

**DOI:** 10.1177/10781552231219003

**Published:** 2023-12-28

**Authors:** Gerard Gurumurthy, Filip Kisiel, Samantha Gurumurthy, Juditha Gurumurthy

**Affiliations:** 1 523364 School of Medical Sciences, 5292 The University of Manchester, Manchester, UK; 2 151626 School of Chemical Engineering and Analytical Science, 5292 The University of Manchester, Manchester, UK; 3 170895 School of Infectious Disease and Immunity, 4615 Imperial College London, London, UK; 4 572204 School of Cancer and Pharmaceutical Sciences, King's College London, London, UK

**Keywords:** Chemotherapy induced thrombocytopenia, thrombopoietin, chemotherapy toxicity, eltrombopag, romiplostim

## Abstract

**Introduction:**

Chemotherapy-induced thrombocytopenia (CIT) is a significant challenge in cancer treatment, often leading to dose reductions and reduced number of cycles. The limited effectiveness of platelet transfusions in managing CIT highlights the need for alternative treatments. Thrombopoietin receptor agonists (TPO-RA), including romiplostim, eltrombopag and avatrombopag, have shown potential in increasing platelet counts in CIT patients, necessitating a comprehensive analysis of their efficacy.

**Methods:**

This meta-analysis followed the Preferred Reporting Items for Systemic Reviews and Meta-analysis guidelines, searching Ovid databases up to 5 October 2023. The primary metric of interest was platelet count changes post-TPO-RA administration in CIT patients.

**Results:**

From the initial 867 studies obtained, 7 studies were selected based on the inclusion criteria. The analysis included 348 patients. A significant association was found between TPO-RA administration and platelet count increase, with a combined-effect increase of 69.52 ± 2.24 × 10^9^/l. Subgroup analysis based on Romiplostim use suggested an increase of approximately 70.11 ± 39.07 × 10^9^/l, while non-Romiplostim TPO-RAs showcased an increase of about 68.09 ± 82.58 × 10^9^/l.

**Conclusions:**

The meta-analysis demonstrates the effectiveness of TPO-RAs in managing CIT. Further research comparing platelet increases across standardised TPO-RA regimens is recommended to refine treatment strategies. This analysis provides valuable insights for clinicians in tailoring CIT treatment using TPO-RAs.

## Introduction

Myelosuppression is a known toxicity associated with chemotherapy.^1–3^ Studies have demonstrated that chemotherapy exerts a substantial impact on cells within the bone marrow microenvironment.^4^ This effect diminishes their capacity to facilitate regular hematopoiesis and may result in damage to the bone marrow, contributing to chemotherapy-induced thrombocytopenia (CIT).^5^ The Common Terminology Criteria for Adverse Events (CTCAE)^6^ v5.0 defines CIT when the platelet count is below 100 × 10^9^/l. While haematopoietic growth factors have been established in the treatment of neutropenia and anaemia, there lacks a defined management strategy in CIT, with platelet transfusions being the most common solution.^7^

Platelet transfusions often exhibit transient effects and are not practical to maintain the levels of thrombocytes through chemotherapy nadir. Patients are therefore at risk of a bleeding disorder and subsequently undertake a dose reduction of their chemotherapy regime to avoid the potential complications of CIT and the need for multiple transfusions.^8,9^ This may negatively impact the goals of their treatment.^2,10^ The limitations in management strategies have resulted in the need for alternative thrombopoietic agents in the context of CIT to reduce multiple platelet transfusions and ensure the completion of the chemotherapy regimens.

Thrombopoietin receptor agonists (TPO-RA), such as romiplostim, eltrombopag and avatrombopag are thrombopoietin agents initially designed for the treatment of immune thrombocytopenia.^11–13^ All three drugs interact with the TPO receptor, c-MpL, encouraging the differentiation and proliferation of megakaryocytes.^14^ Romiplostim is administered subcutaneously and is a recombinant Fc-peptide fusion protein. In contrast, eltrombopag and avatrombopag are orally available drugs that bind to the transmembrane region of c-MpL.^15^ All three drugs have been previously investigated, albeit to various degrees, on their efficacy in raising platelet count in various grades of CIT.^16^ Exploring the platelet count increase of TPO-RA may be beneficial for clinicians when deciding if a patient may benefit from TPO-RA over platelet transfusions. Hence, we undertook a meta-analysis to determine the role of TPO-RA in CIT.

## Methodology

### Search strategy and inclusion criteria

This study was performed in accordance with the Preferred Reporting Items for Systemic Reviews and Meta-analysis. The search was conducted on databases available through Ovid (from inception to 5 October 2023) with the following strategy:

(*Chemotherapy induced thrombocytopenia* OR *CIT* OR *Chemotherapy*) AND (*Romiplostim* OR *AMG 531* OR *Eltrombopag* OR *Avotrombopag* or *Thrombopoietin*) AND (*platelet count* OR *platelet*)

A detailed search strategy is attached to the Supplemental Data 1. The search was limited to articles written in English. Formats such as reviews and editorials were excluded. The primary metric of interest was the increase in platelet count following TPO-RA administration. Therefore, studies that stated platelet count before/after treatment, or stated the overall platelet count increase, were included specifically for the analysis.

### Data extraction

Two reviewers independently extracted the following data that met the inclusion criteria from only the studies that were included in the meta-analysis: mean age, number of patients, type of TPO-RA and dosage, tumour type, chemotherapy regime, platelet count pre-TPO-RA, and the highest platelet count value post-TPO-RA.

### Quality assessment

The Newcastle-Ottawa scale (NOS) was used to assess the quality of the included studies. Both reviewers assessed each study against the NOS and scores greater than 5 were included in the meta-analysis. Publication bias was assessed through Egger's regression test and a funnel plot assessing for symmetry.

### Statistical analysis

Heterogeneity among studies was investigated using I-squared (*I*^2^) test. Random-effect model was used for pooling the results. An *I*^2^ value below 25% is considered indicative of low heterogeneity, a range from 25% to 50% suggests moderate heterogeneity and a value exceeding 50% is seen as high heterogeneity.^17^ A 95% confidence interval (CI) was used as the combined effect measure. Weightage was calculated using a standard method based on the standard error of the estimated effect size of platelet increase. The possible covariates of heterogeneity were investigated by integrating several study characteristics into a meta-regression model. A covariate was identified as a contributing factor to heterogeneity if its inclusion in the model resulted in a reduction in the *I*^2^ value. A sensitivity analysis was also conducted by excluding each study individually and observing its effect on heterogeneity. This comparative approach allows for a more precise understanding of the covariate's impact on the heterogeneity observed across studies.

All significance was examined with Chi-square with the degree of freedom set at k−1 , where *k* was the number of studies in that particular analysis. In addition, a *z*-score was utilised in the meta-regression to compare the significance between sub-group analyses of interest. Significance was set at *p* < 0.05 for both and the analyses were performed using Microsoft Excel (Microsoft Corporation, Washington, USA).

## Results

Eight-hundred sixty-seven studies were initially obtained through the search. After screening and retaining studies relevant to the inclusion criteria, six studies^18–23^ were included in this meta-analysis. One study reported an increase in platelet count in two separate groups. Both groups were deemed to be within the inclusion criteria and were added to the meta-analysis, bringing the total number of studies to seven (Table 1). Note that one of these studies was an abstract submitted to a conference but was deemed to have contained all the relevant data and was therefore included.

**Table 1.** Baseline characteristics of patients and design of included studies.

Study name	TPO-RA	Control group (if any)	Initial dose	Frequency	Duration	Chemotherapy regime (*n*)	Type of cancer (*n*)	No. of subject	Age (range)	Platelet count pre-TPO-RA	Platelet count post-TPO-RA	Platelet increase
Al-Samkari et al. (2021)^18^	Romiplostim	-	3 mg/kg	Weekly	10- 15 weeks	Alkylating agent-based regimen (13) Antifolate (methotrexate or pemetrexed single agent (4) FOLFIRI (14) FOLFOX and other platinum/5-FU doublets (11) Gemcitabine (single agent) (7) Platinum plus gemcitabine (11) Platinum plus anthracycline, etoposide, or pemetrexed (9) Taxane or taxane-likec (single agent) (16) Targeted therapyd (e.g., tyrosine kinase inhibitor) (20) Immunomodulatory (4) FOLFIRINOX or FOLFOXIRI (11) Fluoropyrimidine (single agent) (20) Gemcitabine plus taxane (10) Platinum plus taxane (10) Single-agent platinum, anthracycline, or vinca alkaloid (8) Temozolomide (8) Others (10)	Breast (11) CNS (15) Colorectal (23) Gastroesophageal (18) Genitourinary (2) Gynecologic (11) Head and neck (5) Hepatobiliary (22) Lung (13) Lymphoma (13) Myeloma (7) Neuroendocrine (6) Pancreatic (22) Sarcoma (5)	73	60 (19–85)	66.0 ± 10.0	106.0 ± 25.0	40.0 ± 26.9
Al-Samkari et al. (2021)^18^	Romiplostim	-	3 mg/kg	Intracycle	10- 15 weeks			80	60 (19–85)	54.0 ± 15.0	143.0 ± 50.0	89.0 ± 52.2
Zhu et al. (2021)^19^	Eltrombopag	Untreated Observation	50 mg	Daily	10 days	CHOP or CDOP (23) R-EPOCH (3) R-CODOX-M or R-IVAC (6) CAM (3), VDLP (4) P-Gemox (5) DICE (4) Others (3)	DLBCL (28) T or B lymphoblastic (7) NK/T-cell lymphoma (5) PTCL (3) Burkitt lymphoma (6) Other types (2)	51	49.1 (15–86)	24.0 ± 14.2	130.7 ± 70.6	106.8 ± 72.0
Al-Samkari et al. (2022)^20^	Avotrombopag	Placebo	60mg	Daily	5 days before the primary chemotherapy and for 5 days immediately following the chemotherapy	Gemcitabine, Fluorouracil, Carboplatin, Cisplatin, Anthracycline, or an Alkylating agent (quantity not stated)	Ovarian, bladder, or lung cancer (quantity not stated)	82	61 (38–78)	29.6 ± 13.7	81.6 ± 67.8	51.5 ± 61.9
Gao et al. (2021)^21^	Avotrombopag	-	60mg	Daily	8 weeks	Platinum-based chemotherapy (12) Others (1)	Gastric (2) Breast (2) Endometrial (1) Ovarian (1) Lung (2) Colorectal (4) Gallbladder (1) Nasopharyngeal (1)	13	64 (51–71)	8.7 ± 5.3	55.31 ± 38.8	46.6 ± 39.1
Soff et al. (2019)^22^	Romiplostim	Untreated Observation	2.0 µg/kg	3 weekly	8 weeks minimum, or duration of 2 cycles of chemotherapy	Not stated	Breast (4) GI (8) Lung (2) Sarcoma (1)	15	50 (30–76)	62.0 ± 4.0	141.0 ± 6.0	79.0 ± 7.2
Moufarrij et al. (2023)^23^	Romiplostim	-	Not stated	Not stated	3 weeks	DNA synthesis inhibitor (8) Combination regimen (11) Antibody-mediated (8) Microtubulin target (7)	Breast (27) Ovarian (4) Endometrial (3)	34	55 (45–64)	53.0 ± 28.4	147.0 ± 393.3	94.0 ± 394.4

TPO-RA: thrombopoietin receptor agonist.

### Quality assessment

All seven studies scored five or more points on the NOS scale, suggesting a good overall study quality. Egger's test and funnel plot determined no potential publication bias (*p* > 0.05) (Supplemental Data 2).

### Characteristics of the included studies

**Figure 1.**
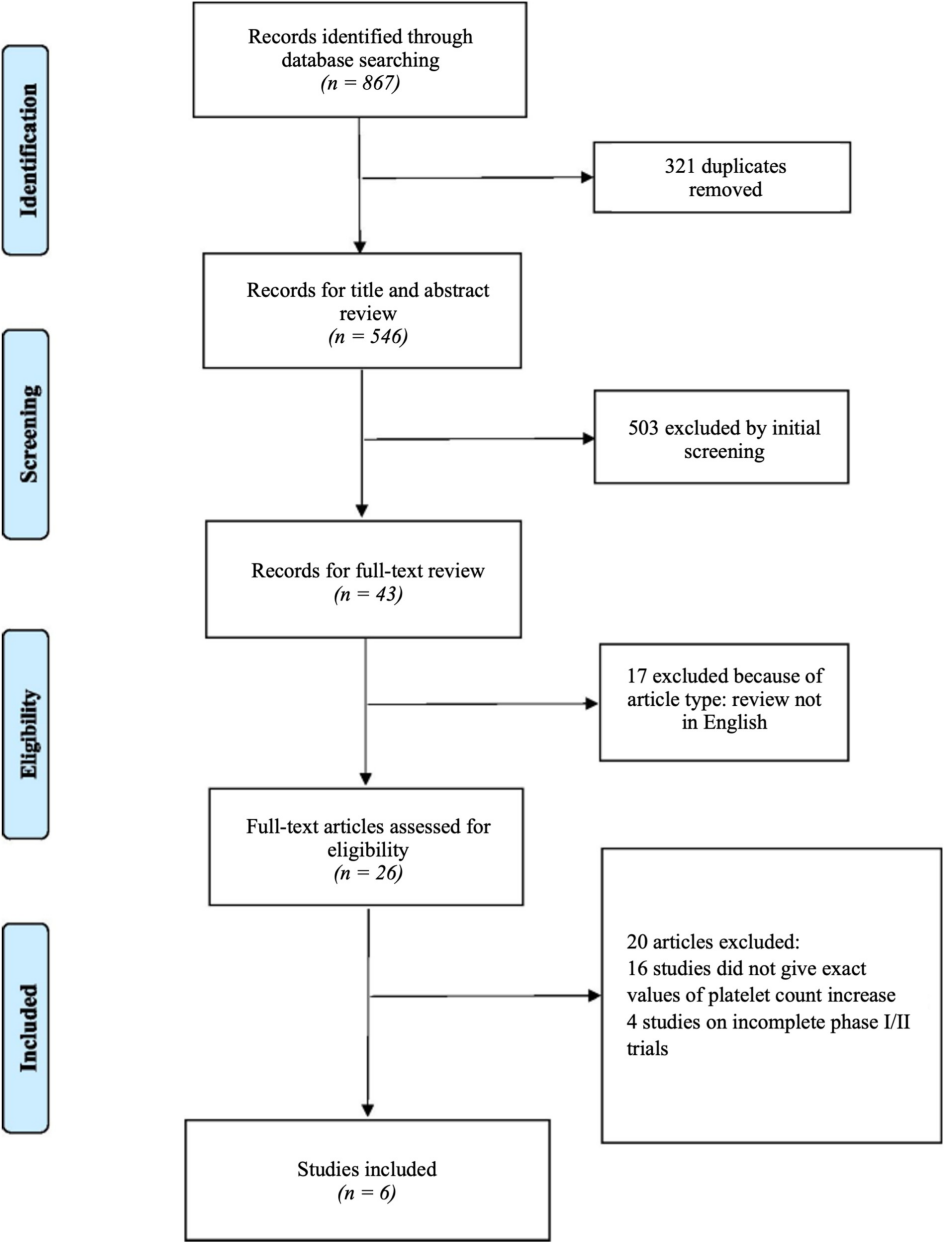
PRISMA flowchart of retuned number of studies from the search strategy and the number of included studies. Note that one study evaluated platelet count increase in two groups, thereby bringing the number of studies in this analysis to seven.

The meta-analysis encompassed a total of seven studies involving 348 patients. The average age of participants across these studies was approximately 56.52 years (range 15–86 years). The extracted data provided insights into the specific TPO-RA used, as well as the pre- and post-platelet count values (Figure 1).

### Correlation between platelet count increase and TPO-RA administration

Our analysis demonstrated a significant association between TPO-RA administration and platelet count increase in patients with CIT. The combined-effect platelet count increase was calculated to be 69.52 ± 2.24 × 10^9^/l. The statistical analysis determined a significant positive correlation between TPO-RA and platelet count increase (*p* < 0.01, *I*^2^ = 96.0%, 95% CI = 67.28, 71.77).

### Subgroup analysis and meta-regression

**Figure 2.**
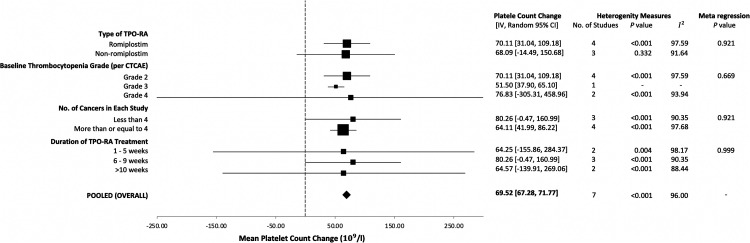
Meta-analysis of the sub-groups and pooled (overall) effect of the association between thrombopoietin receptor agonists (TPO-RA) and change in platelet count. Sources of heterogeneity were explored through meta-regression but failed to reach significance.

A subgroup analysis and meta-regression were performed focusing on the change in post-intervention platelet count (Figure 2). Sources of heterogeneity, specifically the type of TPO-RA, the grade of thrombocytopenia, the different number of cancer types, and the duration of TPO-RA treatment of each study were explored but, failed to reach statistical significance (*p* > 0.05). The sensitivity analysis revealed that the association between the TPO-RA and the increase in platelet count was consistent when the analysis conducted sequentially omitted each study. Focussing specifically on the subgroup analysis on the use of romiplostim, which is the most common TPO-RA investigated for CIT, our analysis highlighted a similar platelet count increase in studies with romiplostim (*n* = 4) at 70.11 ± 39.07 × 10^9^/l compared to studies of non-romiplostim drugs (eltrombopag and avatrombopag, *n* = 3) at 68.09 ± 82.58 × 10^9^/l (Figure 2). *Z*-scores statistical measures indicate that the difference in platelet count increase between the two groups is not significant (*p* > 0.05). It should be noted, however, that a lack of statistical significance does not suggest that the two TPO-RAs are equivalent.

## Discussion

Our primary analysis, encompassing seven studies, revealed a substantial increase in platelet count post-TPO-RA administration, averaging an increase of 69.52 ± 2.24 × 10^9^/l. This result is statistically significant (*p* < 0.01) and underscores the potential of TPO-RAs as an effective intervention for CIT. As the primary goal of TPO-RA in CIT therapy is to avert bleeding, maintaining a platelet count in the range of 50–150 × 10^9^/l is suitable for most patients.^24^ Hence, the derived value in platelet count increase is suggestive that TPO-RA is not only efficacious in achieving therapeutic platelet levels but also in potentially surpassing the minimum threshold required for bleeding prevention. This underscores its viability as a frontline intervention for patients with CIT, providing both a rapid and robust response in platelet elevation. Several studies report a correction of platelet count to more than 100 × 10^9^/l within two to three weeks, which suggests that there might be limited interruptions between chemotherapy cycles.^15,23,24^ Indeed, it has been noted that romiplostim significantly reduced the need for delayed/missed chemotherapy cycles and platelet transfusion, thus ensuring the completion of the chemotherapy regime.^18,19,24^

With regards to the subgroup analysis on romiplostim, the lack of statistical significance (*p* > 0.05) between the platelet count increase in romiplostim and non-romiplostim TPO-RA may be attributed to the difference in starting thrombocytopenia grades/platelet count. Indeed, all four studies involving romiplostim fell within Grade 2 thrombocytopenia (50 to <75 × 10^9^/l) while the non-romiplostim studies fell in Grades 3 and 4. Specifically, the Grade 3 (25 to <50 × 10^9^/l) category, which had a single study representation, exhibited a mean platelet count increase post-TPO-RA administration of 51.50 ± 13.60 × 10^9^/l (95% CI = 37.90, 65.10) while two studies categorised under Grade 4 thrombocytopenia (<25 × 10^9^/l) had a mean platelet count increase post-TPO-RA administration at 76.83 ± 382.14 × 10^9^/l (95% CI = −305.31, 458.96). Hence, it is suggested that the TPO-RAs investigated are efficacious in the setting of CIT.

The meta-regression conducted to assess the factors contributing to the change in post-intervention platelet counts yielded non-statistically significant *p*-values for the covariates investigated (*p* > 0.05). While the type of TPO-RA, baseline platelet counts and sample sizes appeared to account for a portion of the variability among the study outcomes, these associations did not reach the conventional threshold for statistical significance. This lack of statistically significant findings suggests that while these factors may have a potential role in the observed changes in platelet counts, they do not, in isolation, provide a sufficient explanation for the heterogeneity observed across studies. Consequently, these results highlight the potential for other, unmeasured variables to influence platelet recovery post-chemotherapy and underscore the importance of comprehensive modelling that includes a broader range of studies and treatment-related factors. Considering these findings, it is challenging to conclusively determine which group of patients benefit the most and from which specific TPO-RA agents. To draw definitive conclusions, more studies, especially for various CIT severity grades, are needed. Additionally, considering other factors, such as the dose and frequency of TPO-RA, patient demographics and the severity of their condition, could provide more insights.

Nonetheless, the clinical implications of our findings extend beyond the statistical analysis, highlighting the potential of TPO-RAs to contribute to a significant elevation in platelet counts among CIT patients. The ability of TPO-RAs to consistently surpass the minimum platelet threshold required for bleeding prevention is promising, offering a viable alternative to frequent transfusions. Our study supports the individualisation of TPO-RA therapy, encouraging clinicians to tailor treatment based on patient-specific factors such as baseline platelet counts and prior transfusion history.

This analysis is limited by the variability in study designs, patient populations and treatments, which might have contributed to the heterogeneity in the results. The lack of standardisation in dosage and regimen of TPO-RA will contribute to variability in the platelet count increase. The limited number of studies, especially for certain thrombocytopenia grades, means that our conclusions might change with the addition of more data. Studies with fixed TPO-RA dosages and regimens are warranted to provide more conclusive and comparative studies.

## Conclusion

In summary, our meta-analysis underscores the efficacy of TPO-RAs in managing CIT, with both romiplostim and a combination of eltrombopag and avatrombopag demonstrating effectiveness. Patients with higher thrombocytopenia grades seem to benefit more from TPO-RA treatment and transition closer to normal platelet levels post-administration, specifically when using romiplostim for this group. Studies comparing the platelet increases across a standardised regime are needed to provide more definitive values. Nonetheless, data within this analysis will allow clinicians to determine if their CIT patients may benefit from the utilisation of TPO-RA.

## Supplemental Material

sj-pdf-1-opp-10.1177_10781552231219003 - Supplemental material for Role of thrombopoietin receptor agonists in chemotherapy-induced thrombocytopenia: A meta-analysis Supplemental material, sj-pdf-1-opp-10.1177_10781552231219003 for Role of thrombopoietin receptor agonists in chemotherapy-induced thrombocytopenia: A meta-analysis by Gerard Gurumurthy, Filip Kisiel, Samantha Gurumurthy and Juditha Gurumurthy in Journal of Oncology Pharmacy Practice
