# Supplementary material for: Role of thrombopoietin receptor agonists in chemotherapy-induced thrombocytopenia: A meta-analysis
Source: J Oncol Pharm Pract. 2023 Dec 28;31(1):4–11. doi: 10.1177/10781552231219003 (PMC11771093; doi:10.1177/10781552231219003)
Supplement: sj-pdf-1-opp-10.1177_10781552231219003 - Supplemental material for Role of thrombopoietin receptor agonists in chemotherapy-induced thrombocytopenia: A meta-analysis [file sj-pdf-1-opp-10.1177_10781552231219003.pdf]

S/N	Search Term	No. of Results
1	Chemotherapy-induced thrombocytopenia.ti,ab	818
2	CIT.ti,ab	16606
3	Drug therapy/ or chemoradiotherapy/ or chemotherapy, adjuvant/	1284079
4	1 or 2 or	1300502
5	Romiplostim.ti,ab	2455
6	AMG 513.ti,ab	88
7	Eltrombopag.ti,ab	3668
8	Avatrombopag.ti,ab	362
9	Thrombopoeitin/	9978
10	5 or 6 or 7 or 8 or 9	14271
11	Platelet count.ti,ab	91763
12	Platelet count/	86868
13	11 or 12	151082
14	4 and 10 and 13	867

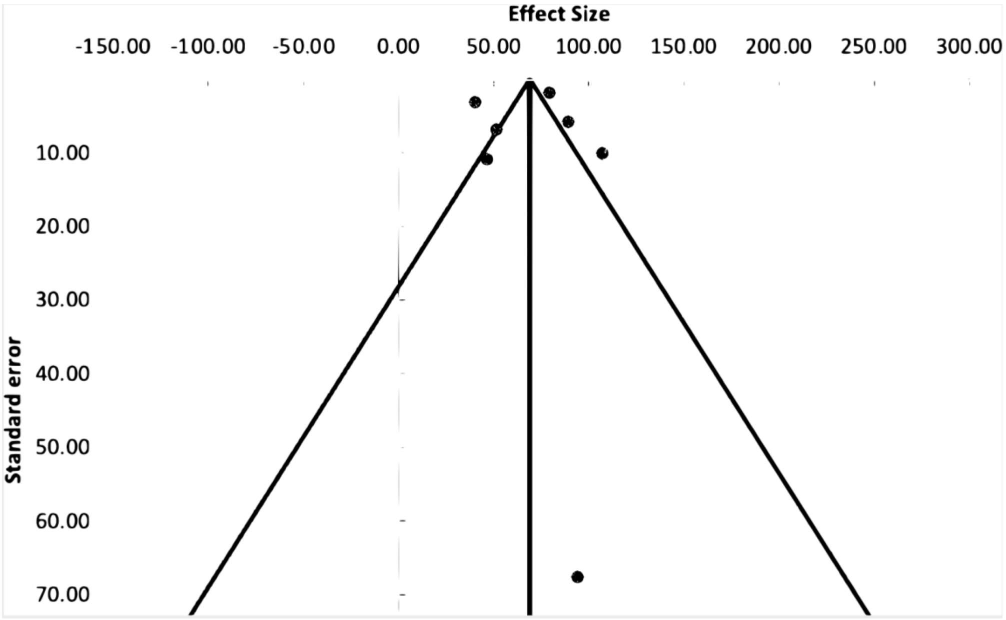

283x174mm (144 x 144 DPI)
